# The development of mature gait patterns in children during walking and running

**DOI:** 10.1007/s00421-020-04592-2

**Published:** 2021-01-13

**Authors:** Margit M. Bach, Andreas Daffertshofer, Nadia Dominici

**Affiliations:** Department of Human Movement Sciences, Faculty of Behavioural and Movement Sciences, Amsterdam Movement Sciences & Institute of Brain and Behavior Amsterdam, Vrije Universiteit Amsterdam, Amsterdam, The Netherlands

**Keywords:** Children, Locomotion, Mechanical energy, Maturity, Clustering

## Abstract

**Purpose:**

We sought to identify the developing maturity of walking and running in young children. We assessed gait patterns for the presence of flight and double support phases complemented by mechanical energetics. The corresponding classification outcomes were contrasted via a shotgun approach involving several potentially informative gait characteristics. A subsequent clustering turned out very effective to classify the degree of gait maturity.

**Methods:**

Participants (22 typically developing children aged 2–9 years and 7 young, healthy adults) walked/ran on a treadmill at comfortable speeds. We determined double support and flight phases and the relationship between potential and kinetic energy oscillations of the center-of-mass. Based on the literature, we further incorporated a total of 93 gait characteristics (including the above-mentioned ones) and employed multivariate statistics comprising principal component analysis for data compression and hierarchical clustering for classification.

**Results:**

While the ability to run including a flight phase increased with age, the flight phase did not reach 20% of the gait cycle. It seems that children use a walk-run-strategy when learning to run. Yet, the correlation strength between potential and kinetic energies saturated and so did the amount of recovered mechanical energy. Clustering the set of gait characteristics allowed for classifying gait in more detail. This defines a metric for maturity in terms of deviations from adult gait, which disagrees with chronological age.

**Conclusions:**

The degree of gait maturity estimated statistically using various gait characteristics does not always relate directly to the chronological age of the child.

**Supplementary Information:**

The online version contains supplementary material available at 10.1007/s00421-020-04592-2.

## Introduction

Running and walking—two everyday types of locomotion in humans—are distinguishable to the naked eye by obvious differences in kinematics and kinetics. When adults run, there is a well-defined flight phase during which none of the legs are in contact with the ground, unlike walking that comprises a double support phase during which both legs are on the ground together. It, therefore, comes as no surprise that the presence or absence of a flight phase is the most commonly used classifier to distinguish walking from running.

Turning to a more biomechanical perspective, walking may be modelled as an inverted pendulum swing: the center-of-mass (CoM) vaults over the stance leg (Alexander 1976) resulting in peaks in the CoM trajectory during mid-stance and an out-of-phase exchange between potential and kinetic energies (Saibene and Minetti 2003). Running, on the other hand, may be modelled as a spring, at least in adults: the stance leg compresses (Carriero et al. 2009; Courtine et al. 2009; Dewolf et al. 2020; Dominici et al. 2012; Fortney 1983; Friedli et al. 2015; Ivanenko et al. 2007a; Phinyomark et al. 2018; Roberts et al. 2017; Van Hooren et al. 2019; Vasudevan et al. 2016; Wenger et al. 2016) resulting in a CoM trajectory with the lowest point at mid-stance and in-phase oscillations of potential and kinetic energies. In adults walking, the amount of saved energy, typically quantified as percentage of the recovered energy, is about 65% at the most optimal speed. In adults running, by contrast, the energy recovery does not depend on speed and fluctuates around 5% (Cavagna et al. 1964, 1976). This observation motivates an alternative and, by now, likewise accepted measure to distinguish walking from running, namely out-of-phase versus in-phase oscillations of potential and kinetic energies as well as the exchange between them.

In children, the ability to walk develops slowly from first independent steps to about 7 years of age, both in terms of mechanical energy and kinematics (Cheron et al. 2001a, b; Dominici et al. 2010; Hallemans et al. 2004; Ivanenko et al. 2004, 2005, 2007b). It seems that the efficient use of the pendulum mechanism during walking develops gradually, the recovery of mechanical energy is lower in toddlers during their first independent steps than in toddlers aged two and up who have more walking experience (Ivanenko et al. 2004), and it is much lower than in adults walking at comparable speeds (Hallemans et al. 2004).

Running in children is not as well researched. Vasudevan et al. (2016) showed that infants are able to take some steps with a flight phase when supported on a treadmill but that their kinematic patterns disagree with adult running. An earlier study in children running revealed instances in which the energy recovery exceeded 11% during slow running (Schepens et al. 1998) and, hence, about twice as high as one may expect for running in adults. Since the corresponding experimental trials were excluded from further analysis, it remains opaque whether or not exaggerated energy recovery values at slow speeds are part of the development of running. In any case, though, a mature and efficient walking pattern seems to develop gradually. This lets us believe that an equivalent gradual change should be visible in the development of running.

Current studies on energetics in children typically assessed over-ground locomotion with one or two strides recorded per trial (Ivanenko et al. 2004; Schepens et al. 2004, 2001; Schepens and Detrembleur 2009). However, over-ground locomotion often introduces more variability in the gait speed than treadmill locomotion. Arguably, speed is easier to correct on the treadmill (Cavagna et al. 1977), but certainly, one can record more strides per participant potentially providing more statistical power in the subsequent analyses. It is for that reason that we adopted this experimental design to answer: (i) how does running on a treadmill develop in children when running is defined as having a flight phase; and (ii) how does this change when defining running as the in-phase oscillations of kinetic and potential energies?

If ‘proper’ running in children is meant to resemble running patterns of adults in some sense, then the development of running implies an increasing degree of gait maturity. Yet, adult gait already comes with substantial variability, raising doubts as to whether identifying the presence of a flight phase or pinpointing phase relationship between the CoM’s kinetic and potential energy will indeed provide a robust means to determine this degree of gait maturity. That is, while (i) & (ii) are relevant questions to ask, one may wonder whether or not the two characteristics they address suffice to quantify the (development of) running in children. In fact, the literature offers a plenitude of kinematic and kinetic parameters and other gait characteristics that might be informative about the gait maturity. We, therefore, supplemented flight phase presence and energy relationship by a large set of parameters that we chose based on previous studies that proved their capacity for categorizing gait patterns (Carriero et al. 2009; Courtine et al. 2009; Dewolf et al. 2020; Dominici et al. 2012; Fortney 1983; Friedli et al. 2015; Ivanenko et al. 2007a; Phinyomark et al. 2018; Roberts et al. 2017; Van Hooren et al. 2019; Vasudevan et al. 2016; Wenger et al. 2016). Without informed pre-selection of parameters, however, such a shotgun approach faces the challenge that parameters may covary and—when combined—do not only yield redundant information but may cause a classification bias. Principal component analysis (PCA) is a common means to remove such covariation and, as such, it comes as no surprise that it has been applied extensively to identify types of locomotion in experimental settings (Courtine et al. 2009; Dominici et al. 2012; Friedli et al. 2015; Wenger et al. 2016). Here, we first applied PCA to rank-reduce our parameter set before clustering the parameters (DeCann et al. 2014; Phinyomark et al. 2015; Sherrill et al. 2005) to classify gait patterns in children by the degree they deviate from gait patterns in adults. With this two-step procedure, we sought to answer (iii) if our ‘blind’ approach allows for pinpointing details of the development of gait in children, and whether it can serve to discriminate between mature and immature locomotion.

## Methods

### Participants

This study included 22 typically developing children aged 2–9 years and 7 young healthy adults as controls for mature patterns, where mature patterns here refer to adult performance. Exclusion criteria were known neurological and developmental diseases. Table 1 provides an overview of the relevant participant characteristics.

**Table 1:** Participant characteristics

	Age (range)	Gender (m/f)	Height (cm)	Weight (kg)
Children	26–106 months	10/12	122 (110–130)	22.5 (18.5–25.7)
Adults	22–28 years	4/3	180 (176–182)	69 (66–78)

Median (25th–75th percentile). Age is the full range

The adult participants and the guardians/parents of the children provided written informed consent in compliance with the Declaration of Helsinki. The children provided assent. The experimental design was approved by The Scientific and Ethical Review Board of the Faculty of Behavioural & Movement Sciences, Vrije Universiteit Amsterdam, Netherlands (File number: VCWE-2016-149R1).

### Setup

The experiment consisted of comfortable walking and running on the treadmill. Every condition was recorded for a minimum of 20 strides where possible and for a maximum of 100 strides.

Participants could familiarize themselves for several minutes on the treadmill during which walking and running were practiced. No set time was imposed. Subsequently, the comfortable speed was determined for both walking and running conditions by starting at a slow speed and increasing in intervals of 0.1 km/h until the participant reported a comfortable speed. In the following, the walking and running conditions refer to the prescribed condition that the participant was asked to perform (i.e., walking and running) during the specific recording.

Children participants wore a full-body climbing harness (CAMP Bambino Full Body Harness, CAMP USA, Colorado) modified to also have a secure attachment point on the back. All participants wore their own shoes for the duration of the experiment.

### Data acquisition

Kinematic data were recorded using an active marker system (Optotrak Motion System, NDI Measurement Sciences, ON, Canada) at 100 Hz. A camera was placed diagonally behind the treadmill on either side and one camera was placed diagonally in front on the right-hand side of the participant. Single markers were attached to the skin overlying the following bony landmarks on the right head of 5^th^ metatarsal, right lateral malleolus (LM), right lateral femoral epicondyle (LE), and right greater trochanter (GT), right and left calcaneus (HE), right and left glenohumeral joint, right and left lateral humeral epicondyle, and right and left ulnar styloid. Kinematics of the right and left upper limbs were of poor quality and did not allow for further analysis. Kinematics could not be recorded in all participants (see Online Resource 3).

Vertical, mediolateral, and forwards ground reaction forces (*F*_v_, *F*_ml_, *F*_f_ GRFs) were sampled at 1 kHz for each belt via the two force plates in the instrumented dual-belt treadmill (Motek Medical BV, Culemborg, the Netherlands).

Foot switches (piezo-resistive pressure sensitive sensors: Zerowire; Cometa, Bareggio, Italy) were placed on the skin on the heel and the head of the first metatarsal underneath the right and the left foot and were secured with tape where necessary. Shoes and socks were placed over the foot switches. The foot switch signals were sampled at 2 kHz. Full-body electromyography recordings were made but not analyzed here. Kinematics, ground reaction forces, and foot switch data were synchronized. At the end of the recording session, anthropometric measurements were taken for every participant. These included mass ($m$) and stature of the participant as well as the lengths of the main body segments.

### Data analysis

#### Flight and double support phases

Step events (heel strike and toe-off bilaterally) were determined based on the kinetic and kinematic data. The vertical ground reaction forces (${F}_{v}$) were pre-processed with a Savitzky-Golay polynomial filter (third order, 121 samples; Savitzky and Golay 1964). We defined heel strike (HS) and toe-off (TO) events as the first samples crossing a fixed threshold (mean(*F*_v_)/10). First and last HS and TO were excluded for further analysis to avoid transients. The HE markers in the vertical direction served to detect step events from the kinematics (Roerdink et al. 2008). The foot switch detection was based on a simple ‘on/off’ algorithm. All events were manually inspected and events were added or removed when needed (e.g., when dragging/jumping). Events were primarily detected based on the* F*_v_, but we supplemented with events based on kinematic and foot-switch detections whenever single foot GRF data were missing or did not allow for event detection. All relevant data were re-sampled to 1 kHz for this application. From the step events, we determined double support and flight phases as well as the corresponding means and standard deviations over all strides per participant and condition. We also computed the walking Froude (Alexander and Jayes 1983) for all participants and conditions based on the treadmill speed and leg length using:

$$Fr \, = \,\frac{{v^{2} }}{g\cdot l}$$

#### Potential and kinetic energies

The combined forces from the right and left force plates of the treadmill served to estimate the kinetic (*E*_k_) and potential energy (*E*_p_) of the CoM in the sagittal plane, following Cavagna (1975); Ivanenko et al. (2004); Saibene and Minetti (2003); Schepens et al. (2004); Schepens and Detrembleur (2009). For full calculations see Online Resource 1. In brief, the kinetic energy *E*_k_ was estimated based on the CoM’s velocity in the vertical and the forward directions. Here, we would like to note that changes of kinetic energy in the medio-lateral direction are usually much smaller than those observed in the vertical and forward directions (Tesio et al. 1998; Tesio and Rota 2019), and that the lateral work can be assumed less than 10% of total work. That is, lateral components can be considered negligible (Schepens and Detrembleur 2009). The potential energy *E*_p_ was determined via the CoM’s position in the vertical direction by integrating the corresponding velocity. Then, we estimated the Pearson correlation coefficients *r *between *E*_k_ and *E*_p_ for each stride to quantify the degree of in-phase and out-of-phase oscillations of the energies.

To quantify the amount of mechanical energy that can be saved via a pendulum mechanism (see Introduction) we determined the relative recovered mechanical energy as (Cavagna et al. 1976):

$$R\, = \,1\, - \,\frac{{W_{{{\text{ext}}}} }}{{W_{{\text{f}}} \, + \,W_{{\text{v}}} }}$$

where the external work (*W*_ext_) was based on the sum of (*E*_k_ + *E*_p_)-increments over a stride and the work in forward and vertical directions (*W*_f_ and *W*_v_) on the sum of increments of the forward and vertical CoM energies, respectively (Cavagna et al. 1976).

#### PCA and clustering

Based on the kinetics and right-side kinematics, numerous spatiotemporal, kinetic, and kinematic parameters were calculated that provided a comprehensive quantification of gait features. In total 93 parameters were determined for every participant when kinematic measurements were available (*n* = 18 participants; 13 children and 5 adults). The parameters can be split into themes that represent modalities of gait. To build on the findings of the ability to run with a flight phase and have in-phase oscillations of potential and kinetic energies during running, we supplemented these parameters with additional temporal features (in total 9 parameters), additional features of the pendulum/swing mechanisms (in total 11 parameters), limb endpoint trajectories (12 parameters), stability measures (3 parameters), segmental and joint angles (21 parameters) and velocities (9 parameters), kinetics (6 parameters), intra-limb coordination (2 parameters), intersegmental coordination (14 parameters), and interlimb coordination (6 parameters). By including parameters from several strides per participant, we implicitly incorporated the variance across strides as this is a common measure for gait variability. For a detailed list of parameters see Online Resource 2. We normalized the parameters that were directly related to the size of the participant to body-size/body-weight (e.g., step length, step height, vertical force; see Online Resource 2 for details). All the parameters were combined in a [(number of participants × condition × number of strides) × number of parameters] matrix [1530 × 93] and z-scored along the first dimension prior to PCA. As outlined above, PCA primarily served to rank-reduce the parameter matrix, which eliminates parameter covariations and, by this, allows for an unbiased classification via conventional clustering (see below). We selected the leading three principal components (PCs) as they turned out to suffice for our classification purposes (Courtine et al. 2009; Dominici et al. 2012; Friedli et al. 2015; Phinyomark et al. 2015). To which degree the different 93 parameters influenced the first three PCs can be given by the corresponding $\mathrm{loadings }= u\cdot \sqrt{\lambda }$, where $u$ denotes the eigenvector of a PC and $\lambda$ its eigenvalue. We considered a parameter a strong contributor to a PC when its loading exceeded the 95% confidence threshold ${\mathrm{CI}}_{95} = 1.96/\sqrt{n}$, with  $n$=93.

Finally, we applied hierarchical clustering. In doing so, we first built a dendrogram or cluster tree (Milligan 1980; Murtagh and Contreras 2011; Xu and Wunsch 2005) using average links (unweighted pair group method with arithmetic mean) based on the correlation distances across the 3D reduced parameter set (we also tested other distance measures, like Euclidean and Mahalanobis distances, but none of them yielded comparably proper clusters). The dendrogram was thresholded based on the cophenetic correlation coefficients (CCC; Sokal and Rohlf 1962) and, for comparison, also by visual inspection, with the latter focusing on both categorization of walking versus running and classification of mature and immature locomotion. To distinguish mature from immature locomotion, we computed the average pairwise correlation distance from every participant belonging to a distinct walking cluster to the adults walking and from every participant belonging to a distinct running cluster to the adults running. Put differently, that distance measures gait maturity with the adult gait pattern as reference.

### Statistics

We assessed the influence of age on the presence of a flight phase (FP) during running, as well as the influence of age and condition on the presence a double support (DS) phase. For this, we used two linear regression models across both children and adults, the first one with FP as response variable and age as predictor, the second one with DS as response variable and age, condition, and the interaction between the two as predictors. For both models we considered  $p<\hspace{0.17em}$0.05 statistically significant.

We quantified the age-dependence of the correlation coefficients $r$ and of the relative recovered energy $R$ by least squares fitting exponential functions $a\cdot e^{{ - \frac{1}{\tau (age - \gamma )}}} \, + \,b$, where τ was the time constant, and a, b, γ three constants, and report their explanatory power, in terms of adjusted *R*^2^-values, unless specified otherwise.

## Results

Although 29 participants were included in the analysis on the mechanical energies of the CoM, only 18 participants were included in the analysis of the effects of kinematic and kinetic parameters on distinguishing mature from immature gait and walking from running. All participant characteristics, as well as the numbers of strides included in each part of the analysis, can be found in Online Resource 3. It is worthwhile adding that the minor differences between stride numbers relate to the quality of the data varying between data sets. The youngest participants that we recorded (< 50 months of age) were all locomoting with handhold from the experimenter or parent/guardian. We confirmed that this did not affect the kinetics post-recording.

### Flight and double support phases

**Fig. 1:**
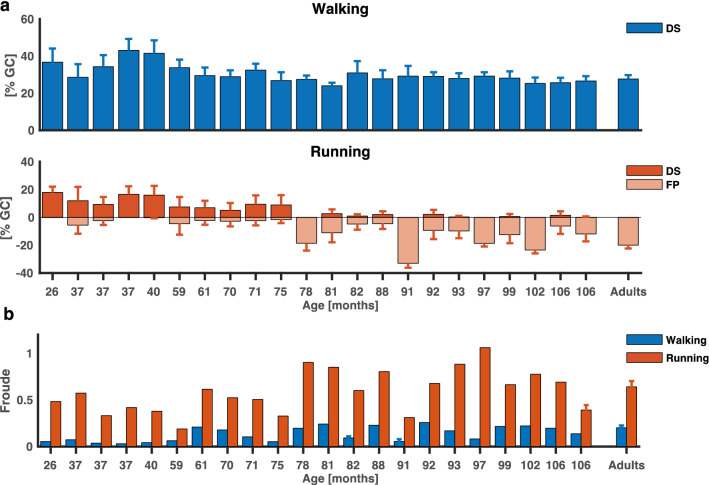
Temporal patterns during walking and running and normalized speed. **a** Double support phase (positive numbers) and flight phase (negative numbers) expressed as a percentage of the gait cycle (mean ± SD) for walking (upper panel) and running (lower panel), as a function of age (months-rounded to the nearest whole integer) for each child participant and adults as a grand average. **b** Normalized speed expressed as the Froude value (*v*^2^/*g·l*) for each participant and condition (walking in blue and running in red). Error bars signify standard deviations between participants for adults and differences between trials for the walking condition of the participants of 82 and 91 months and running condition for the second participant of 106 months

We expressed FP and DS relative to the gait cycle (Fig. 1a). For the running condition, a FP was present in some participants and the linear regression revealed a significant effect of age ($p$ < 2 × 10^–16^), i.e., FP increased with age. DS revealed main effects of both age and condition ($p$ < 2 × 10^–16^, $p$ = 0, respectively), and it decreased for running. We also found an interaction effect ($p$ = 5.8 × 10^–6^) as summarized in Table 2. The normalized speeds given as Froude values differed between conditions for all participants; see Fig. 1b.

**Table 2:** Linear regression of the effects of age and condition on double-support and flight phases

	Factor	Estimate	SE	${\varvec{t}}$	${\varvec{p}}\boldsymbol{ }$-value
**DS**	Intercept	31.03	0.26	117.50	0
	Age	−0.01	0.00	−9.57	< 2 × 10^–16^
	Condition_running	−24.40	0.38	−64.35	0
	Age:condition_running	−0.01	0.00	−4.54	5.8 × 10^–6^
$\mathbf{F}\mathbf{P}$	Intercept	4.63	0.38	12.24	< 2 × 10^–16^
	Age	0.06	0.00	25.62	< 2 × 10^–16^

*DS* double support, *FP* flight phase, *SE* standard error, $t$ *t*-statistics

**Fig. 2:**
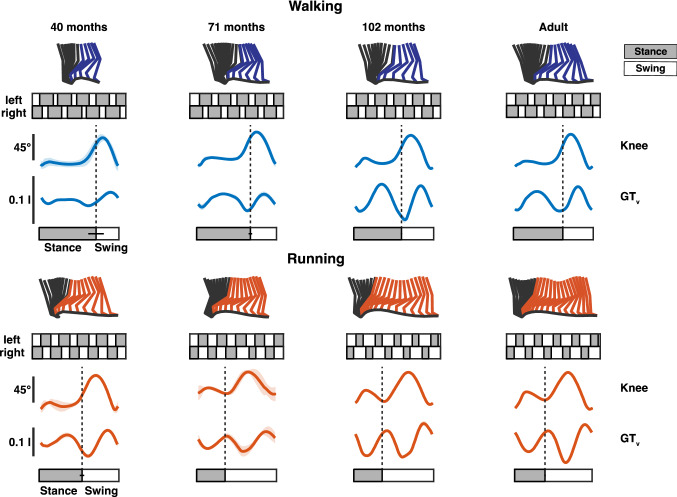
Kinematics during walking and running. Stick figures of representative strides of four representative participants during walking (upper panel) and running (lower panel). The black parts of the stick figures correspond to stance phase and the colored to the swing phase (blue for walking; red for running). Below, five representative strides are presented for each participant for left and right leg. Ensemble averages (± SD of five gait cycles) of knee joint angle and vertical hip displacement (GT_v_) for each participant and condition. Gait cycle bars represent mean stance and swing duration for each participant with the horizontal black bar representing the standard deviation between strides. GT_v_ is expressed in relative units (normalized by the limb length *l*)

The corresponding stick figures, vertical hip displacements, and knee joint angles of four representative participants are presented in Fig. 2. For all participants, the vertical GT displacement (GT_v_) was maximal during mid-stance of the load-bearing leg, adhering to the double-peaked profile of the pendular mechanism of the CoM during walking. During running, the GT_v_ was minimal during mid-stance of the load-bearing leg, suggesting a spring leg behavior of running. However, this was only present in the three oldest participants. In the youngest participants during running, GT_v_ was maximal at mid-stance. In Fig. 2, GT_v_ and the knee joint angle for five strides for each of the displayed participants show a less pronounced pattern in the youngest participants compared to the adult, which suggests a more immature gait pattern in the younger participants and a mature one for the adult participant.

### Potential and kinetic energies

**Fig. 3:**
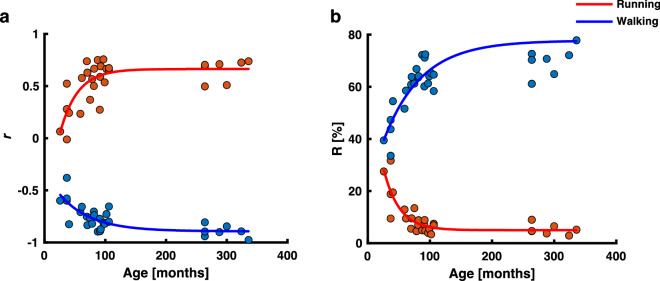
Effects of the mechanical energy of the CoM on age. **a** The correlation coefficient $r$ between *E*_k_ and *E*_p_ as a function of age for walking (blue) and running (red). There is an exponential relationship between age and $r$ for both walking and running. **b** The relative recovered energy *R* as a function of age for walking (blue) and running (red). There is an exponential relationship between $R$ and age for both walking and running

We found a moderate exponential relationship between age and the correlation coefficients $r$ for walking and running (*R*^2^ = 0.53, *R*^2^ = 0.51, respectively; Fig. 3a), while the relative recovered energy $R$ was strongly correlated with age for both walking and running (*R*^2^ = 0.59, *R*^2^ = 0.70, respectively; Fig. 3b).

### Shotgun and clustering

**Fig. 4:**
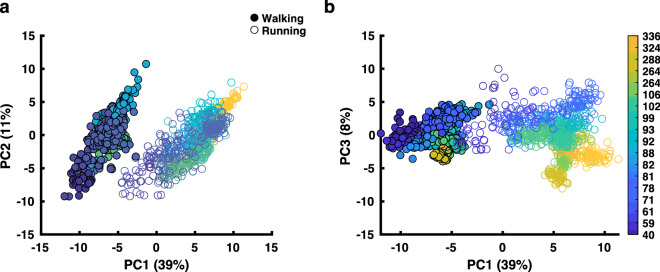
Principal component analysis (PCA) outcomes for walking and running. **a** The outcome of the PCA in PC1-PC2 space. **b** The outcome of the PC1-PC3 space. Each dot represents a stride and the color-coding refer to the age in months of the participants. The filled circles are the prescribed walking condition and the un-filled circles are the prescribed running condition

The first three PCs accounted for 57% of the total variance of the data while this might be considered a low proportion in conventional PCA, one has to realize that we z-scored the input variables which let us consider three PCs to cover a sufficient portion of data variance. The scatterplots in Fig. 4 illustrate the separation between the prescribed walking and running patterns (filled and unfilled markers, respectively) with clear correlations illustrated in the PC1/PC2 plane. The loadings associated with these three PCs revealed that all parameters except for three were significantly larger than the 95% confidence interval, and thus uniformly influenced the variance in the data. The three exceptions were parameters 75, 76, and 82 (cf., Online Resources 2 and 4).

The average linkage and correlation distance had a CCC of 0.92. There were no clear distinctions between the number of clusters when analyzing dissimilarity values. However, visual inspection of the dendrogram indicated that either four or eight clusters adequately represented the original data (see Online Resource 5). A Calinski Harabasz stopping rule (Milligan and Cooper 1985) applied for 1–10 clusters confirmed this split, at least in parts, as it revealed two of the four clusters. Since we aimed for distinguishing mature from immature patterns as well as walking from running patterns, we continued with the four clusters identified visually.

**Fig. 5:**
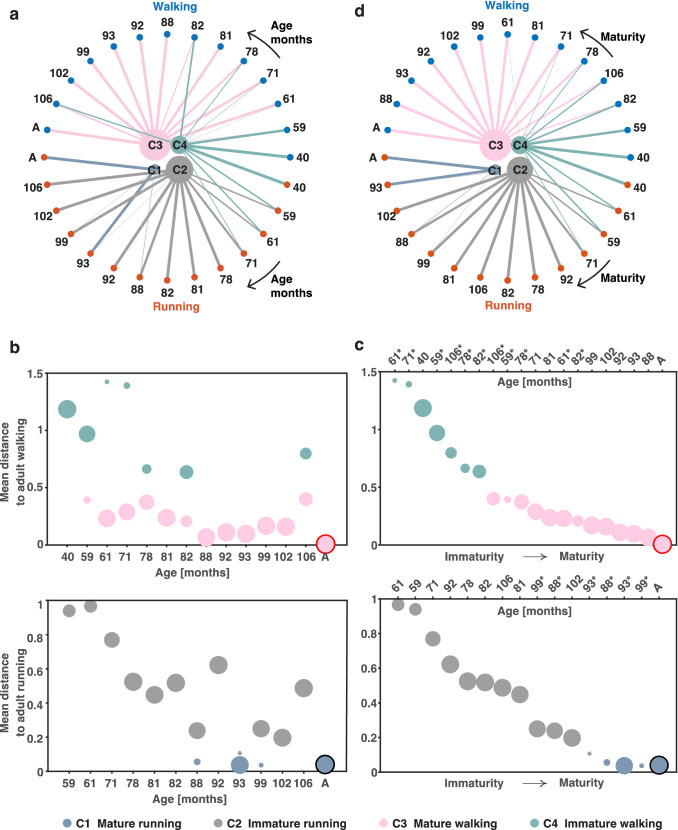
Clustering output. **a** Output of clustering ordered based on age (months), with the youngest participant on the right side and the adults (A) on the left side for walking (blue circles) and running (red circles). The size of the clusters (C1-C4) depends on the amounts of strides belonging to each cluster, similarly the thickness of the lines connecting each cluster with a participant depends on the percentage of data from each participant belonging to that cluster. **b** Calculated average pairwise correlation distance to the mature walking patterns of the adults (A) (upper panel) and to the mature running patterns of the adults (A) (lower panel) as a function of age. **c** Calculated average pairwise correlation distance to the mature walking patterns of the adults (A) (upper panel) and the mature running patterns of the adults (A) (lower panel) as a function of gait maturity. The upper axis in both plots represents the age of the participants in months (rounded to nearest whole integer). Note that the increase in age is not monotonic as it is a function of gait maturity (immature from left going to mature on right). Note also that the lower axis in both plots is not in units of the correlation distance (which is shown on the y-axis) but set to arbitrary values (indices of sorting); that is, the seeming exponential decay should not be interpreted as such. Color notation is the same as in a), C4 represents immature walking, C3 represents mature walking, C2 represents immature running and C1 represents mature running. The size of the circles depends on the amounts of strides belonging to each cluster. **d** Output of clustering based on maturity with the least mature patterns on the right side and the most mature (adults: A) on the left side. For a full overview of the percentage of strides belonging to each cluster, see Online Resource 6

In Fig. 5, each of the clusters is represented with the relation every participant had to them. We expected adults to have a mature walking and running pattern and, accordingly, we grouped them together as a single node (indicated as ‘A’ in Fig. 5). The thickness of the lines represents the percentage of strides, larger than 5%, belonging to a certain cluster. Every cluster is plotted in an individual color. Cluster 1 contained the adults running and 94.7% of the running strides from the participant aged 93 months. Cluster 2 contained between 74.6% and 100% of all prescribed running strides from the participants aged 71 to 106 months bar the participant of 93 months (5.3% of the strides from this participant belonged to cluster 2), together with around 50% of the prescribed running strides from the participants of 62 and 59 months. Cluster 3 covered between 42 and 100% of the prescribed walking strides from the participant aged 62 months to the adults and 16.1% of the walking strides from the participant of 59 months. Finally, cluster 4 contained around 50% of the prescribed running strides from participants of 59 and 62 months, 25% of the prescribed running strides from the participants of 71 months and all prescribed running and walking strides from the participant of 40 months, together with some walking strides from older participants, most importantly, around 80% of walking strides from the participant of 59 months, around 50% of the walking strides from the participants aged 82, and 40% of the walking strides of the participant of 106 months (see Online Resource 6 for further details on the spread of strides into every cluster). As shown in Fig. 5a, the younger children were grouped in separate clusters from the adult participants. The average pairwise distance to the adult patterns for walking and running separately, i.e., our measure for gait maturity is depicted in Fig. 5b. Obviously, there was no directed relationship between our measure of gait maturity and the participants’ age. To illustrate this further in Fig. 5c, we ordered participants based on their respective distance to the mature pattern of the adults depending on whether their strides fall into the walking or running clusters, but here we also included the corresponding ages on the top x-axes. The participant of 40 months is only present in the walking clusters as the strides belonging to the prescribed running conditions are clustered with the walking strides of the other participants. Some participants are present twice as their strides fall into more clusters (see above). In Fig. 5d, the order of the participants has been re-arranged following the maturity order in Fig. 5c. When a participant had strides falling into two clusters, they were ordered based on the position in which most of their strides belong to.

## Discussion

The aim of this paper was to investigate the development of running on a treadmill. Gait classification traditionally relies on the presence of a flight phase or the display of in-phase oscillations of kinetic and potential energies during running. Expectedly, both measures have their limitation in quantifying subtle changes in gait patterns. Hence, we supplemented them by a substantial set of alternative gait characteristics and followed a statistics-based classification of walking and running and the development thereof.

Not all children were able to run with a flight phase. Their running patterns clearly differed from that of adults, but also from (their own) walking. As such, their prescribed running should not be classified as walking. We found exponentially saturating changes in the correlation between kinetic and potential energies and the total amount of recovered mechanical energy, implying there were in-phase oscillations of kinetic and potential energies during running and out-of-phase oscillations during walking. On top of that, our cluster analysis revealed the absence of a direct relationship between chronological age and maturity of the (prescribed) walking and running patterns in children aged 40–106 months.

### Flight and double support phases

Running can be defined as having a flight phase. We showed that young children who are asked to run on a treadmill at comfortable speeds are not able to do so. This might be interpreted as they are in fact not running. At first glance, the gait patterns remind one of walking, but a closer look reveals that they are not walking either. It seems that children learning to run make use of what could be called a “walk-run strategy” that contains both double support and flight phases. The (relative) duration of the double support phase in running, is of course not comparable to that seen in walking. Interestingly, this also extends to the in-phase and out-of-phase oscillations of kinetic and potential energies.

### Potential and kinetic energies

We already mentioned in the introduction that Schepens et al. (1998) studied running in children aged 2–16 years. In that study all trials were excluded in which the relative energy recovery *R* exceeded 11% as they were deemed not to be running trials. This is unfortunate as our finding support the idea that in the learning period the gait is a mix of a walking and a running pattern. That is, *R* may occasionally exceed 11% during running, in our case in six participants. When incorporating the correlation coefficients $r$, however, it is still possible to distinguish between conditions for all participants as the potential energy oscillates out-of-phase for the walking conditions ($r$ being negative) and in-phase for the running conditions ($r$ being positive). In fact, the two types of locomotion (walking and running) are different in speed for all participants in our study (see Fig. 1b and Online Resource 1), and despite the young participants sometimes running with double support phase, this is still different from the double support phases observed in the walking condition.

Yet, we have to admit that the overall findings in the energetics, with the exponential relationships between $R$ and $r$ and age are mostly influenced by the youngest participants. It seems that the energetics are not fine-grained enough to distinguish between older children and adults and thus will not reveal how running matures from children older than 3.5 years to adults.

### Shotgun and clustering

Chronological age and gait maturity of treadmill locomotion are not directly related in children aged 3.5–9 years. Maturity of one type of locomotion is also not directly linked to that of the other type of locomotion and as such, a child can display mature walking, but not mature running and vice versa. We defined gait maturity as the pairwise correlation distance from adult patterns and thus used the mean of the adult walking and running patterns, respectively, as a reference for mature patterns. This allowed us to rank participants based on their individual distance to the mature patterns of walking and running, separately.

These results appear more complete, indeed, when compared to those on flight phases and energetics only. We are, therefore, inclined to argue that a shotgun approach with proper pattern classification can provide additional insight in the development of running in children. In an earlier study, Phinyomark et al. (2015) already showed that two distinct kinematic running patterns in adults running can be identified combining PCA and clustering analysis on separate kinematic waveforms. In our eight-cluster analysis, we found one adult with a separate running pattern from all other participants and this finding could be related to differences in the running pattern (see Online Resource 5). However, we here considered the adult group as a single group as, despite differences between adults they display a ‘mature’ pattern, and as such we chose the four-cluster result as the main result.

We ‘blindly’ selected 93 parameters, with which we succeeded to categorize gait patterns and classify their change in the course of development. The obvious next step is to identify which of these parameters have significant contributions to the classification. One can in fact isolate subsets of the parameter by their contribution to principal components (see, e.g., Kaptein et al. 2014). In doing so, we found that the temporal parameters (such as flight phase/double support phase) and pendulum/swing mechanisms (e.g., the oscillations of kinetic and potential energies) do greatly influence PC1. However, others were also adding to it, like leg/joint velocities and limb endpoint velocities. That is, when it comes to the time course of development, many if not all these parameters seem to covary, a fact that of course also extends to PC2 and PC3. From the composition of PC1 one may conclude that—albeit important—the mere presence of flight phase and in-phase oscillations of potential and kinetic energies does not suffice to distinguishing walking from running. More information is needed to pinpoint the (type of) gait pattern and define its degree of maturity. Yet, one has to realize that in our gait classification PCA primarily served for rank reduction followed by hierarchical clustering. Isolating relevant parameters in the space spanned by three principal components for their contribution to the correlation distance based hierarchical clustering is clearly less trivial. Here we hope for future work to provide rigorous methods to determine which specific parameters play a role for each of the clusters; more advanced statistical models like genetic algorithms may help with this. Only if this or alternative methods will succeed, can our shotgun approach not only ‘describe’ the change of gait patterns, but may serve as unbiased means to determine which parameters are crucial for this change.

### Limitations and choices

In the current data set participants of around 4 years of age are absent due to recruitment or measurement issues. This leaves a relatively large gap of 19 months between the child of 40 months and the child of 59 months. We do not expect our outcomes to change qualitatively when that gap is filled, but without a doubt it can provide further information on the maturity of locomotion in the younger children.

The type of locomotion referred to throughout this paper is the prescribed locomotion and this means that despite asking the children to run and either them or their parent/guardian confirmed it as running, they might not have been able to run as they would have over ground. Despite this potential limitation, we are positive that –in our experiments– the prescribed running patterns were not like those one would expect for walking or even fast walking.

Our participants were walking and running on a treadmill at a constant comfortable speed during the whole trial, with the advantage that the amounts of strides analyzed for each condition varied between 15 and 76 strides (Online Resource 3). This is in contrast to most other studies on the mechanics of locomotion, where participants walk or ran over ground and thus did not record more than two or three steps per trial with up to ten trials per participant (Ivanenko et al. 2004; Schepens et al. 2004, 1998; Schepens and Detrembleur 2009) amounting to a 10–15 strides per participant. Moreover, it is more difficult to control the speed of the participant when locomoting over ground compared to on a treadmill and as such more fluctuations in the speed of the participant are expected. Speed fluctuations are important to account for when analyzing the energetics during average locomotion (Cavagna et al. 1977).

A final note on data ‘pre’-processing: Prior to performing PCA, data were z-scored along the first dimension. The normalization of parameters across strides results in the adult values not skewing the PCA and cluster analysis in terms of amplitude. When looking at Fig. 4, it seems that the variability between and within participants was not larger in the older children than in the adults, arguably due to the normalization. Variability within participants hence appears an unlikely cause for larger correlation distances from the young children to the adults in our clustering approach. Moreover, not all parameters were normalized to body-size/body-weight (see Online Resource 2) prior to the z-scoring, PCA, and subsequent clustering. The ones that were normalized directly relate to the size of the participant (e.g., step length, step height, vertical force), whereas for example joint and segmental angles are already considered dimensionless (see, e.g., Hof 1996). One may argue, however, that (almost) all the parameters might have been influenced by both the participants’ size and the speeds performed. Yet, there were several instances of a single participant being split into more than one cluster, while maintaining the same speed. We hence do not believe, that speed or body size were influencing factors in our cluster results.

Kinematic and kinetic parameters are influenced by neural factors and vice versa. A recent comprehensive review on the neural circuitries and biomechanics of walking and running in development (Dewolf et al. 2020) showed that running patterns mature during childhood but that the underlying mechanisms are still not thoroughly investigated. Here, we give some insights into the underlying kinematics and kinetics of this development. However, we did not investigate the muscular components as part of this study. We know from adults that the muscle activity patterns differ between adults walking and running and that there is a reduction in the duration of contraction with age for both the medial gastrocnemius muscle in walking for typically developing children (Cappellini et al. 2016; Tirosh et al. 2013), as well as in the thumb adductor during pinching movements (Dayanidhi et al. 2013). These findings suggest that the immature locomotor patterns found in this study could be correlated to increased contraction time. A recent study in children with cerebral palsy showed that it was possible to change their kinematic gait patterns without influencing their selective motor control (Booth et al. 2019). However, whether this also applies to typically developing children should be confirmed with further analysis of the muscle activity signals. Another recent study investigating muscle activity patterns during running in preschoolers and adults with different training experience revealed substantial developmental and training-related plasticity suggesting a long-term reorganization to satisfy the biomechanical changes and functional requirements of locomotion (Cheung et al. 2020).

## Conclusion

Clustering revealed that there is no direct agreement between chronological age and gait maturity in young children walking and running when comparing their gait patterns to those of adults. When learning to run, young children employ a “walk-run-strategy”. This strategy provides the ability to run with a combination of strides with double support and flight phase and yields in-phase oscillations of potential and kinetic energies.

## Supplementary Information

Below is the link to the electronic supplementary material.

Supplementary file1 (PDF 772 KB)

## Data Availability

The data that support the findings of this study are available upon request from the corresponding author (ND). The custom-made code used to analyze data for this publication is also available upon request from the corresponding author (ND).

## Abbreviations

CCC: Cophenitic correlation coefficient
CoM: Center-of-mass
*E*_k_: Kinetic energy
*E*_p_: Potential energy
*F*_v_: Vertical ground reaction force
GT: Greater trochanter
GT_v_: Vertical displacement of GT
HE: Calcaneus marker (heel)
HS: Heel strike
LE: Lateral epicondyle
LM: Lateral malleolus
PC: Principal component
PCA: Principal component analysis
*r*: Pearson correlation coefficient between *E*_k_ and *E*_p_
SD: Standard deviation
SE: Standard error
*t*: t-statistics
TO: Toe-off
VM: Fifth metatarsal
