# Supplementary material for: The development of mature gait patterns in children during walking and running
Source: Eur J Appl Physiol. 2021 Jan 13;121(4):1073–85. doi: 10.1007/s00421-020-04592-2 (PMC7966230; doi:10.1007/s00421-020-04592-2)
Supplement: Supplementary file 1 — Supplementary file1 (PDF 772 KB) [file 421_2020_4592_MOESM1_ESM.pdf]

The development of mature gait patterns in children during walking and running

Margit M. Bach, Andreas Daffertshofer, Nadia Dominici

Department of Human Movement Sciences, Faculty of Behavioural and Movement Sciences, Amsterdam Movement Sciences & Institute of Brain and Behavior Amsterdam, Vrije Universiteit Amsterdam, Amsterdam, The Netherlands.

Corresponding author: Nadia Dominici, n.dominici@vu.nl

Online Resource 1

The following illustrates the steps taken in order to calculate the relevant energetic parameters. First, the acceleration (a) of the center-of-mass (CoM) was calculated in the sagittal plane:

$$a_f = \frac{F_f}{m} \tag{1}$$

$$a_v = \frac{F_v}{m} - g \tag{2}$$

Where, F = Ground reaction forces, f = forward, v = vertical, m = body mass, g=9.81ms$^{-2,}$ gravitational acceleration.

Next, we calculated the velocity of the CoM in the sagittal plane:

$$v_i = \int a_i \, dt + c_i \tag{3}$$

Where *i* denotes either the forward, or vertical direction, c_i is the integration constant. The integration constants were found by calculating the mean speed of the GT marker on the assumption that GT is close to the CoM and so the corresponding velocity is equal to the mean speed of the CoM.
The instantaneous kinetic energy E_k of the CoM was then defined as:

$$E_k = \frac{1}{2} m \, v_f^2 + \frac{1}{2} m \, v_v^2 \tag{4}$$

Kinetic energy comprises of the vertical and forwards components computed in (3). The instantaneous potential energy E_p of the CoM was given as:

$$E_p = mg \int v_v \, dt + c \tag{5}$$

The integration constant *c* is arbitrary and was taken equal to 0.
The total mechanical energy of the CoM E_{tot} was the sum of E_k and E_p waveforms over a stride:

$$E_{tot} = E_k + E_p \tag{6}$$

The positive external work W_{ext} was calculated as the sum of increments of E_{tot} over a stride (Cavagna et al. 1976). As well as, the forward work W_f and the vertical work W_v were computed as the sum of increments of E_f and E_v, respectively*:*

$$E_f = \frac{1}{2} m v_f^2 \tag{7}$$

$$E_v = E_p + \frac{1}{2} m\, v_v^2 \tag{8}$$

Finally, the percentage recovery R introduced by Cavagna et al. (1976) estimate the ability to save mechanical energy and was determined as:

$$R = 1 - \frac{W_{ext}}{W_f + W_v} \tag{9}$$

Since W_{ext} may depend on stride length and on the participants' anthropometry we normalized it via

$$W_{\text{ext}} \to \widehat{W}_{\text{ext}} = \frac{1}{m \cdot d} W_{\text{ext}}$$

where, m denotes the participant's body mass and d the stride length.

As a measure of the variability between strides we computed the standard deviation of r denoted by $\sigma(r)$. There was a moderate exponential relationship between age and $\sigma(r)$ of both walking and running ($R^2 = 0.67$, $R^2 = 0.57$, respectively; Fig. OR1a), whereas W_{ext} did not correlate with age (R2 = 0.16, R2 = 0.02, walking and running, respectively; Fig. OR1b).

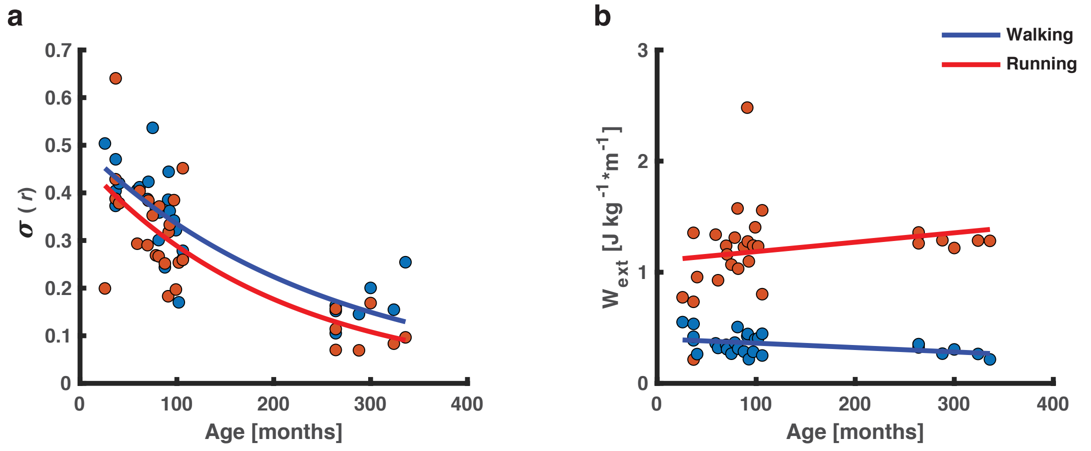

Fig. OR 1 *Effects of variability of the correlation between mechanical energies of the CoM and external work on age a) The variability of the correlation between E_k and E_p, $\sigma(r)$, as a function of age for walking (blue) and running (red). There is an exponential relationship between age and $\sigma(r)$ for both walking and running. d) The external work as a function of age for walking (blue) and running (red). There is no linear relationship between the external work and age for walking and running*

Effect of speed

Children of different sizes and adults ran and walked at different speeds and thus we correlated the measures to the Froude number, i.e. to the dimensionless speed. The Froude number is a measure for speed normalization across participants of different sizes. It is given by (Alexander and Jayes 1983)

$$Fr = \frac{v^2}{g \cdot l}$$

here, v denotes the speed, g = 9.81ms^{-2} is the gravitational constant and l is the participant's leg length and measured as thigh (GT–LE) plus shank (LE–LM) length.

For the analysis of r, R, $\sigma(r)$, and W_{ext}, linear and exponential curves were fitted as appropriate to investigate the relationships with age and the normalized speed (walking Froude number), respectively. The adjusted R^2-value will be reported unless specified otherwise.

The spread in speeds was not (entirely) age dependent, as can be observed in the overlap of the lightest and darkest colors dots in Fig. OR2. The correlation coefficient r between E_k and E_p during walking and running was weakly correlated to the Froude number (R^2 = 0.31 and R^2 = 0.22, respectively; Fig. OR2a). There was no relationship between $\sigma(r)$ and the Froude number for running (R^2 = -0.02), although there was a weak to moderate relationship between $\sigma(r)$ and the Froude number during walking (R^2 = 0.36 Fig. OR2b). Furthermore, we found a moderate relationship between R and walking (R^2 = 0.58), while we could not identify any relationship between the R and the Froude number during running (R^2 = 0.133; Fig. OR2c). Likewise absent was a relationship between W_{ext} and the Froude number during walking and running (R2 = 0.14, R2 = -0.00, respectively; Fig. OR2d).

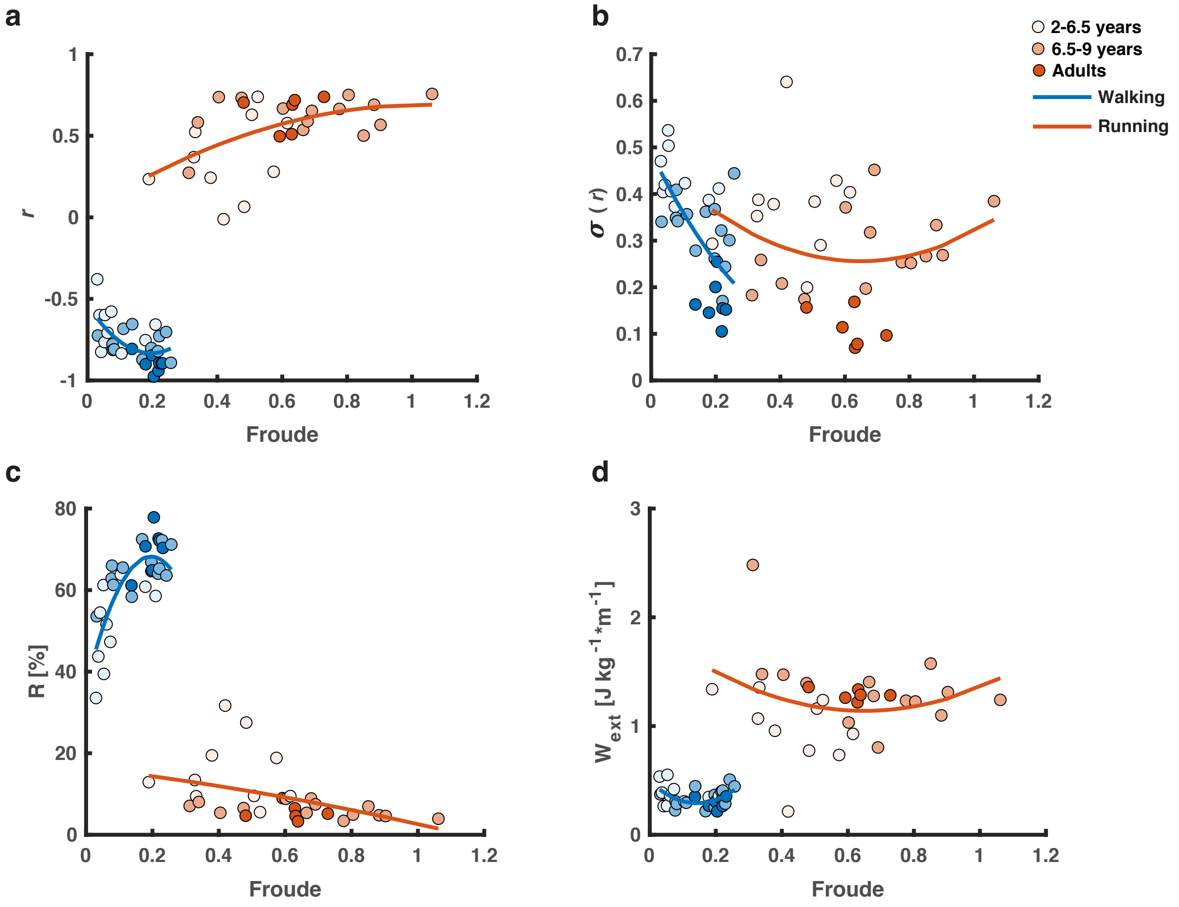

Fig. OR2 *Effects of the mechanical energy of the CoM on dimensionless speed (Froude number). a) The correlation coefficient* r *between* E_k *and* E_p *as a function of the Froude number for walking (blue) and running (red). There is a second order polynomial relationship between the Froude value and* r *for both walking and running. b) The variability of the correlation* r *between* E_k *and* E_p*,* $\sigma(r)$*, as a function of the Froude number for walking (blue) and running (red). There is a relationship between* $\sigma(r)$ *Froude for walking, but not for running. c) The percentage recovery* R *as a function of Froude for walking (blue) and running (red). There is a relationship between* R *and Froude for walking, but not for running. d) The external work as a function of Froude for walking (blue) and running (red). There is no relationship between* W_{ext} *and Froude for walking and running. For all panels in the figure applies that the color-gradient refers to the age of the participant and the lightest colors are the youngest participants where the darkest colors are the adults*

In adults, the transition from walking to running is abrupt (Lamoth et al. 2009), but whether this was the case in our study, or whether the transitions were as smooth as those found in infants (Vasudevan et al. 2016) or in adults, e.g., in a reduced gravity simulation (Labini et al. 2011), remains opaque. Future studies on the walking-running gait transitions combined with clustering in children may further elucidate this.

Hallemans et al. (2004) found that the maximal recovery during walking was around 40% in toddlers aged 12-18 months. The range we find in children aged 2-9 years is between 35%-70%, and we see an increase with age. In our study we find a relationship between the percentage recovery and the dimensionless speed. There is a direct link between the smaller stature (and thus slower speed) and the ability to recover energy when walking on a treadmill. Participants were walking at comparable dimensionless speeds.

We find no relationships between external work and age or dimensionless speed during running and a limited relationship during walking indicating that some participants are locomoting at an optimal speed. The lack of relationship was also shown when comparing normal weight adolescents with their overweight counterparts running at the same speed (Taboga et al. 2012), however this is in contrast to the work presented in the introduction (Hallemans et al. 2004; Schepens et al. 2004; Van de Walle et al. 2010). We do not find any relationship for running due to the elastic energy which is not accounted for in external work in running (Cavagna et al. 1977; Saibene and Minetti 2003). However, we would have expected some relationship between the external work and age and Froude value for walking. The finding can be related to the differences between over ground and treadmill running.

Alexander RM, Jayes AS (1983) A Dynamic Similarity Hypothesis for the Gaits of Quadrupedal Mammals J Zool 201:135-152

Cavagna GA, Heglund NC, Taylor CR (1977) Mechanical work in terrestrial locomotion: two basic mechanisms for minimizing energy expenditure Am J Physiol 233:R243-261 doi:10.1152/ajpregu.1977.233.5.R243

Cavagna GA, Thys H, Zamboni A (1976) The sources of external work in level walking and running J Physiol 262:639-657 doi:10.1113/jphysiol.1976.sp011613

Hallemans A, Aerts P, Otten B, De Deyn PP, De Clercq D (2004) Mechanical energy in toddler gait. A trade-off between economy and stability? J Exp Biol 207:2417-2431 doi:10.1242/jeb.01040

Labini, F. S., Ivanenko, Y. P., Cappellini, G., Gravano, S., & Lacquaniti, F. (2011). Smooth changes in the EMG patterns during gait transitions under body weight unloading. Journal of neurophysiology, 106(3), 1525–1536. https://doi.org/10.1152/jn.00160.2011

Lamoth, C. J., Daffertshofer, A., Huys, R., & Beek, P. J. (2009). Steady and transient coordination structures of walking and running. Human movement science, 28(3), 371–386. https://doi.org/10.1016/j.humov.2008.10.001

Saibene F, Minetti AE (2003) Biomechanical and physiological aspects of legged locomotion in humans Eur J Appl Physiol 88:297-316 doi:10.1007/s00421-002-0654-9

Schepens B, Bastien GJ, Heglund NC, Willems PA (2004) Mechanical work and muscular efficiency in walking children J Exp Biol 207:587-596 doi:10.1242/jeb.00793

Taboga P, Lazzer S, Fessehatsion R, Agosti F, Sartorio A, di Prampero PE (2012) Energetics and mechanics of running men: the influence of body mass Eur J Appl Physiol 112:4027-4033 doi:10.1007/s00421-012-2389-6

Van de Walle P, Desloovere K, Truijen S, Gosselink R, Aerts P, Hallemans A (2010) Age-related changes in mechanical and metabolic energy during typical gait Gait Posture 31:495-501 doi:10.1016/j.gaitpost.2010.02.008

Vasudevan EV, Patrick SK, Yang JF (2016) Gait Transitions in Human Infants: Coping with Extremes of Treadmill Speed PLoS One 11:e0148124 doi:10.1371/journal.pone.0148124

Online Resource 2

Computed gait parameters from kinematic and kinetic recordings. Parameter numbers refer to variables in PCA. Normalized parameters are denoted with square brackets [].

Abbreviations: Param: Parameter; Norm: normalization; l, leg-length; FS, foot strike; TO, toe-off; SE, end-of-swing; AP, anterior-posterior; ML, medio-lateral; w, body weight; SS, single support; d, stride length (1D); Min, minimum; Max, maximum; Amp, amplitude; $\boldsymbol{u}$, eigenvector; elev, elevation.

PARAM	DETAILED EXPLANATION	UNIT [NORM]
**TEMPORAL FEATURES**		
1	Stride duration	s
2	Froude velocity	
3	Stance duration	s
4	Percentage swing duration	% GC
5	Percentage stance duration	% GC
6	Percentage double support	% GC
7	Percentage flight phase	% GC
8	Stride length (1D)	[1/l]
9	Stride length (3D)	[1/l]
**LIMB ENDPOINT (VM) TRAJECTORY**		
10	Step length	[1/l]
11	Step height	[1/l]
12	Maximum backward position	[1/l]
13	Minimum forward position	[1/l]
14	Maximum velocity during swing	m/s
15	Relative timing of max velocity during swing	% GC
16	Acceleration at swing onset	m/s^2
17	Endpoint velocity	m/s
18	Orientation of velocity vector at swing onset	rad
19	Position of ankle with respect to hip at FS	[1/l]
20	Position of ankle with respect to hip at TO	[1/l]
21	Position of ankle with respect to hip at SE	[1/l]
**STABILITY**		
22	Lateral displacement of foot during swing	[1/l]
23	Step width (AP)	[1/l]
24	Step width (ML)	[1/l]
**JOINT AND SEGMENTAL ANGLES**		
25	Thigh elevation angle (Min)	deg
26	Shank elevation angle (Min)	deg
27	Foot elevation angle (Min)	deg
28	Main-leg elevation angle (Min)	deg
29	Thigh elevation angle (Max)	deg
30	Shank elevation angle (Max)	deg
31	Foot elevation angle (Max)	deg
32	Main-leg elevation angle (Max)	deg
33	Knee joint angle (Min)	deg
34	Ankle joint angle (Min)	deg
35	Limb abduction (Min)	deg
36	Knee joint angle (Max)	deg
37	Ankle joint angle (Max)	deg
38	Main-leg abduction (Max)	deg
39	Thigh elevation angle (Amp)	deg
40	Shank elevation angle (Amp)	deg
41	Foot elevation angle (Amp)	deg
42	Main-leg elevation angle (Amp)	deg
43	Knee joint angle (Amp)	deg
44	Ankle joint angle (Amp)	deg
45	Main-leg medio-lateral angle (Amp)	deg
**LEG/JOINT ANGULAR VELOCITY**		
46	Main-leg velocity (Min)	deg/s
47	Knee joint velocity (Min)	deg/s
**LEG/JOINT ANGULAR VELOCITY**		
48	Ankle joint velocity (Min)	deg/s
49	Main-leg velocity (Max)	deg/s
50	Knee joint velocity (Max)	deg/s
51	Ankle joint velocity (Max)	deg/s
52	Main-leg velocity (Amp)	deg/s
53	Knee joint velocity (Amp)	deg/s
54	Ankle joint velocity (Amp)	deg/s
**KINETICS**		
55	Mean AP force during stance phase	N
56	Mean ML force during stance phase	N
57	Mean vertical force during stance phase	[1/w]
58	Mean AP force during SS phase	N
59	Mean ML force during SS phase	N
60	Mean vertical force during SS phase	[1/w]
**INTRA-LIMB COORDINATION**		
61	Correlation limb-arm AP direction	
62	Phase relationship between limb-arm	
**INTERSEGMENTAL COORDINATION**		
63	Percentage of variance (1st u)	
64	Percentage variance (2nd u)	
65	Percentage variance (3rd u)	
66	Projection of 1st u on thigh axis	
67	Projection of 1st u on shank axis	
68	Projection of 1st u on foot axis	
69	Projection of 2nd u on thigh axis	
70	Projection of 2nd u on shank axis	
71	Projection of 2nd u on foot axis	
72	Projection of 3rd u on thigh axis	
73	Projection of 3rd u on shank axis	
74	Projection of 3rd u on foot axis	
75	Area of the gait loop	deg^2
76	Ratio of left to right leg cycle duration	
**INTERLIMB COORDINATION**		
77	Phase difference hip and knee elev. angles	
78	Phase difference foot and shank elev. angles	
79	Max r (thigh and shank elevation angles)	
80	Max r (shank and foot elevation angles)	
81	Max r (knee and ankle joint angles)	
82	Max r (ankle and foot joint angles)	
**PENDULUM/SPRING MECHANISM**		
83	Amplitude of vertical hip displacement	m
84	Amplitude of ML hip displacement	m
85	Forwards work W_f	[1/w]
86	Vertical work W_v	[1/w]
87	External work W_{ext}	[1/d·w]
88	Recovery in percentage (R)	%
89	Amplitude kinetic energy E_k	[1/w]
90	Amplitude potential energy E_p	[1/w]
91	Amplitude total energy E_{tot}	[1/w]
92	Maximal correlation between E_k and E_p	
93	Lag between E_k and E_p	% GC

Online Resource 3

Number of strides analyzed for each child participant (CH) and the adults (AD) as a group for each condition in each analysis. A total of 29 participants were included in the analysis on the mechanical energies of the CoM (Energetics), and 18 participants were included in the analysis of the effects of kinematic and kinetic parameters (PCA-Clustering). Adult data is presented as the median (25th-75th percentile). For the adults, n= 7 for the energetic analysis and n = 5 for running PCA-clustering analysis and n = 4 for the walking analysis for the PCA-clustering.

SUBJ	AGE (MONTHS)	ENERGETICS		PCA-CLUSTERING	
		Walking	Running	Walking	Running
**CH1**	26	24	57	-	-
**CH2**	37	21	24	-	-
**CH3**	37	35	61	-	-
**CH4**	37	22	17	-	-
**CH5**	40	15	17	13	16
**CH6**	59	26	32	26	31
**CH7**	61	32	29	31	29
**CH8**	70	40	40	-	-
**CH9**	71	71	38	71	38
**CH10**	75	14	30	-	-
**CH11**	78	64	86	64	83
**CH12**	81	22	23	22	23
**CH13**	82	26	26	26	26
**CH14**	88	29	31	29	31
**CH15**	91	15	40	-	-
**CH16**	92	30	34	30	34
**CH17**	93	76	56	76	56
**CH18**	97	17	28	-	-
**CH19**	99	56	62	56	62
**CH20**	102	68	45	68	45
**CH21**	106	40	45	-	-
**CH22**	106	71	12	71	12
**A1-A7**	Adults	39 (32.0-55.5) n=7	44 (28.5-50.0) n=7	53 (39.0-58.0) n=4	50 (41.9-59.5) n=5

Online Resource 4

The loadings related to the three PCs. The color-coding refers to the loading with a darker color meaning a higher loading for that factor and PC. For an overview of the parameters, see Online Resource 2.

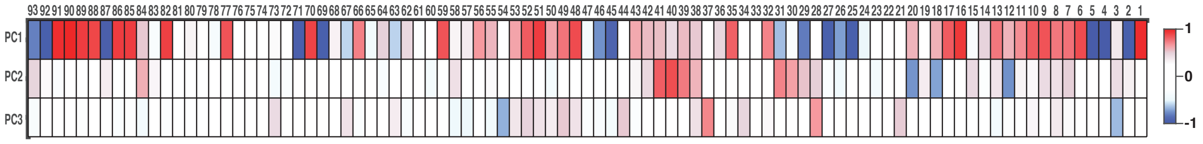

Online Resource 5

Dendrogram showing the split into eight clusters. The clusters one to four (or cluster one and two in the four-cluster solution) are the clusters containing mostly the prescribed running condition (see Online Resource 6). Clusters five to eight (or clusters three and four in the four-cluster solution), are the clusters containing mostly the prescribed walking condition. The y-axis on the figure is a measure for the distance between clusters as measured with the correlation distance. The taller the links between two leaf nodes, the longer the leaf nodes (or clusters) are situated from each other in the 3D space.

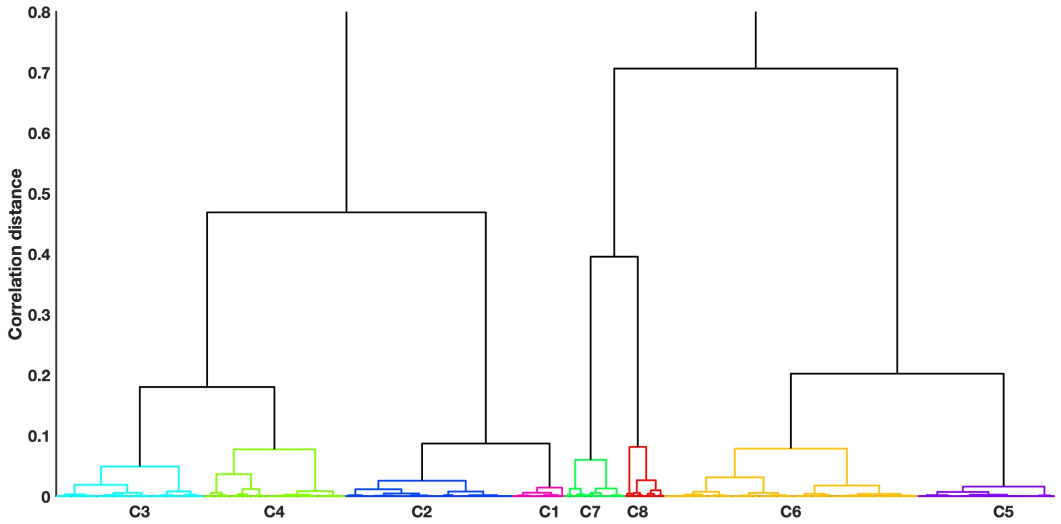

Clustering output for eight clusters. Ordered based on age (months – rounded to the nearest whole integer), with the youngest participant on the right side and the adults (A) on the left for walking (blue circles) and running (red circles). The size of the clusters (C1-C8) depend on the amounts of strides belonging to each cluster, similarly the thickness of the lines connecting each cluster with a participant depend on the percentage of data from each participant belonging to that cluster. For a full overview of the percentage of strides belonging to each cluster, see Online Resource 6.

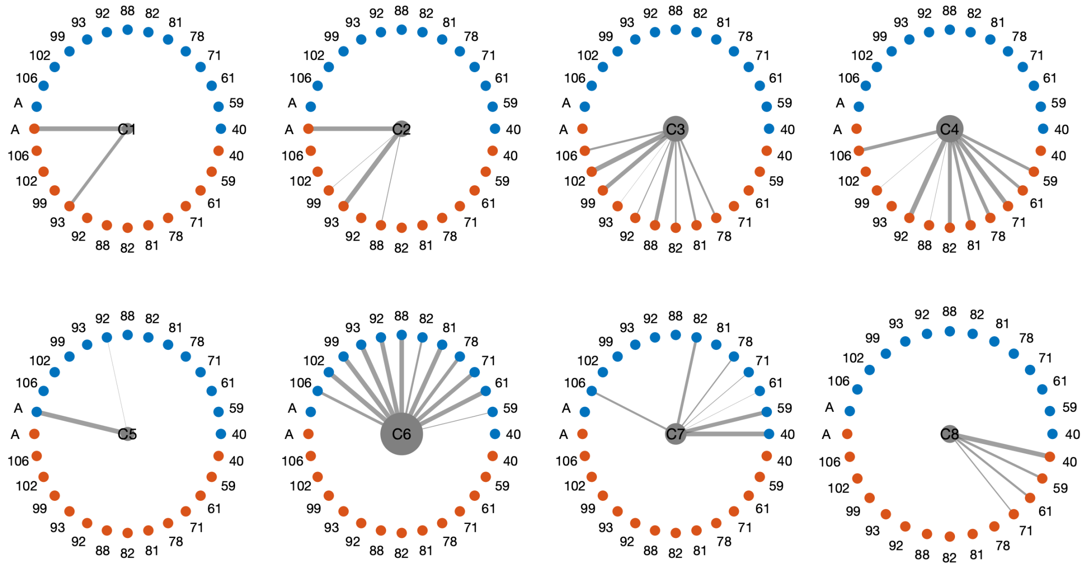

Online Resource 6

The percentage of strides from each participant that belongs to each cluster for the four-cluster solution (left) and eight-cluster solution (right). Numbers in red are the percentage of strides smaller than five percent and are not considered in the figures presenting the cluster analysis.

AGE	4 CLUSTERS				8 CLUSTERS							
	C1	C2	C3	C4	C1	C2	C3	C4	C5	C6	C7	C8
**WALKING**												
**40**				100.0							100.0	
**59**			16.1	83.9						16.1	83.9	
**61**			93.1	6.9						93.1	6.9	
**71**			86.8	13.2						86.8	13.2	
**78**			72.3	27.7						72.3	26.5	1.2
**81**			95.7	4.3						95.7	4.3	
**82**			42.3	57.7						42.3	57.7	
**88**			100.0						3.2	96.8		
**92**			100.0						5.9	94.1		
**93**			100.0							100.0		
**99**			100.0						1.6	98.4		
**102**			100.0						2.2	97.8		
**106**			58.3	41.7						58.3	41.7	
**A1**			100.0						100.0			
**A2**			100.0						100.0			
**A3**			100.0						100.0			
**A4**			100.0						98.2	1.8		
**A5**												
**RUNNING**												
**40**				100.0								100.0
**59**		50.0		50.0				50.0				50.0
**61**		51.6		48.4				51.6			3.2	45.2
**71**		74.6		25.4			1.4	73.2				25.4
**78**		100.0					40.6	59.4				
**81**	4.5	95.5				4.5	45.5	50.0				
**82**		100.0					34.6	65.4				
**88**	13.8	86.2				13.8	79.3	6.9				
**92**		100.0					20.0	80.0				
**93**	94.7	5.3			13.2	81.6	5.3					
**99**	7.1	91.1		1.8		7.1	85.7	5.4			1.8	
**102**	1.5	98.5				1.5	98.5					
**106**		100.0					42.3	57.7				
**A1**	100.0					100.0						
**A2**	97.3	2.7				97.3	2.7					
**A3**	100.0					100.0						
**A4**	96.6	3.4				96.6	3.4					
**A5**	100.0				100							
